# Oncologist-Reported Barriers and Facilitators to Offering Cancer Clinical Trials to Their Patients

**DOI:** 10.3390/curroncol31060230

**Published:** 2024-05-28

**Authors:** Brenda S. Castillo, Leigh Boehmer, Janelle Schrag, Alexandra Howson, Randall Oyer, Lori Pierce, Nadine J. Barrett, Carmen E. Guerra

**Affiliations:** 1 Department of Medicine, Perelman School of Medicine, University of Pennsylvania, Philadelphia, PA 19104, USA; cguerra@pennmedicine.upenn.edu; 2 Association of Community Cancer Centers, Rockville, MD 20850, USA; lboehmer@accc-cancer.org; 3 Whitman-Walker Institute, Washington, DC 20009, USA; schrag.js@gmail.com; 4 Thistle Editorial LLC., Snoqualmie, WA 98065, USA; alex@alexhowson.com; 5 Ann B. Barshinger Cancer Institute, Penn Medicine Lancaster General Health, Lancaster, PA 17601, USA; randall.oyer@pennmedicine.upenn.edu; 6 Rogel Comprehensive Cancer Center at the University of Michigan, Ann Arbor, MI 48109, USA; ljpierce@med.umich.edu; 7 Duke Clinical and Translational Science Institute and Duke Cancer Institute, Durham, NC 27701, USA; njbarret@wakehealth.edu; 8 Abramson Cancer Center, University of Pennsylvania, Philadelphia, PA 19104, USA; 9 Leonard Davis Institute, University of Pennsylvania, Philadelphia, PA 19104, USA

**Keywords:** qualitative research, barrier, facilitator, clinical trials, cancer, disparities

## Abstract

NCCN guidelines indicate that cancer clinical trials (CCTs) are the best management for patients with cancer. However, only 5% of patients enroll in them. We examined oncologists’ perceived barriers and facilitators to discussing CCTs. This qualitative study was part of the ASCO-ACCC Initiative to Increase Racial and Ethnic Diversity in Clinical Trials. Barriers and facilitators at the system, trial, provider, and patient levels were examined. To achieve triangulation, patient encounters were reviewed using chart-stimulated recall (CSR) methods, thereby obtaining a valid assessment of physician performance. Ten oncology providers participated in this study. Nine were oncologists, and one was a clinical research coordinator; five were female; four were White; three were Asian; and three were Black. Barriers to offering CCTs were a lack of trial availability; ineligibility; a lack of knowledge; assumptions about patient interest, benefits, or harms; patient’s disease factors; and negative attitudes. Facilitators of offering CCTs were a physical space to discuss trials; greater trial availability; a systematic approach to offering trials; patient factors; patients seeking trials; a lack of comorbidities; patients being younger in age; patients being aware of, asking about, or hearing of trials from their surgeon; and higher levels of altruism. Many of the cited barriers are addressable with the cited facilitators. A larger study is needed to generalize and validate these findings.

## 1. Introduction

The National Comprehensive Cancer Network (NCCN) states that a clinical trial is the best treatment for any patient [1]; however, approximately 5% of patients with cancer enroll in cancer clinical trials (CCTs) [2,3,4,5,6]. Low CCT accrual impacts the ability to investigate new cancer therapies. Approximately 20% of CCTs fail to be completed due to inadequate accrual [7]. The cost of clinical trials for oncology drugs approved by the FDA in 2015–2017 was $37.1 million per trial [7]. Therefore, low accrual in CCTs poses a threat both financially and scientifically. Kitterman et al. examined the impact of the termination of clinical studies because of low enrollment [8]. They found that one out of every three terminated clinical studies at Oregon State Heath and Science University had enrolled zero or one participant. With this profound impact to oncology treatment development, along with economic repercussions, it is imperative to investigate the barriers and facilitators that affect CCT accrual and that investigators can utilize to improve CCT participation.

Physician recommendation is one of the strongest factors patients consider when deciding to participate in CCTs. When physicians offer patients a CCT to participate in, 55% of them agree to do so, regardless of their race [9]. There are limited data on the oncologist’s perceived barriers and facilitators to the recommendation of CCTs. Understanding their perspective is essential to design interventions to increase their recommendation of CCTs. Previous studies have examined physicians’ perceived barriers and facilitators through surveys [10]. One study that examined actual physician recommendations of CCTs, suggested that rates of recommendation are low (38%) and inconsistent, but did not explore the actual barriers and facilitators [11]. Though there have been studies that discuss oncology providers’ perceived barriers and facilitators to clinical trial enrollment, such studies have focused on pediatric cancers [12,13,14]. Additionally, studies have yet to validate perceived barriers and facilitators to the discussion of CCTs with adult patients with cancer.

We performed qualitative interviews with oncologists and elicited their perceived barriers and facilitators to the recommendation of CCTs. In addition, to achieve triangulation, oncologists reviewed their patient encounters using “Chart-Stimulated Recall” (CSR) to identify if a trial was recommended or not why or why not, and to determine the barriers and facilitators of CCT discussion. By utilizing CSR, we were able to identify the actual barriers and facilitators of the discussion of CCTs between oncology providers and their adult patients with cancer.

## 2. Materials and Methods

This qualitative study was approved by the Institutional Review Board of the Western Copernicus Group (WCG). It was determined to not constitute human subject research and thus received a Quality Improvement Exemption from the WCG IRB. We conducted in-depth, semi-structured interviews with academic and community-based oncologists practicing in the U.S. Study participants answered a national joint invitation from the American Society of Clinical Oncology (ASCO) and the Association of Community Cancer Centers (ACCC) in 2021. The invitation asked for volunteers for a project to test a new unconscious bias training and site self-assessment tool developed in a joint initiative by both organizations titled the “ASCO-ACCC Initiative to Increase Racial Diversity in Cancer Clinical Trials”. This parent study aimed at designing trainings and tools tailored for CCTs’ research teams and to support the enrollment of diverse participants in CCTs [15,16,17]. Members of these two national organizations were invited to apply to participate in the Just Ask unconscious bias training, the site self-assessment to support equity, diversity, and inclusion in clinical trials, or both. Seventy-five nationwide sites responded to the invitation and were then selected to participate. Each participating site was asked to identify up to three program participants to participate in the studies, ideally one from each of the following roles: study investigator/enrolling clinical staff member, research staff member, and non-research staff member who engages with clinical trials. Participants’ characteristics such as type of practice, size of practice, geographic region, and type of community were obtained.

All study participants who responded to the parent study invitation were enrolled in the Just ASK Increasing Diversity in Cancer Clinical Research: An ACCC-ASCO Training Program. A purposive sample from that group was then invited to participate in the current study. Participants were purposively sampled to reflect a diversity of gender, race, ethnicity and geographic location.

Interviews were conducted by a trained researcher in July and August of 2021. Interviews were audiotaped, transcribed verbatim, and lasted approximately 60 min. Participants did not receive remuneration.

The interview guide was designed using an adaptation of the Unger et al. framework that divides the trial decision-making pathway into structural, trialal, provider, and patient levels [4]. System refers to structural factors such as the workspace and workforce. The absence of an available trial and trial eligibility represents a trial barrier.. If the patient is eligible, the physician may discuss and offer trial participation. Only then does the patient decide whether to participate. Eligible patients may not enroll due to either not being asked or declining when they are asked.

Appendix A includes the questions that were used in the interview guide. The interview guide was formulated to explore the four levels of barriers and facilitators to the discussion of CCTs. Unstructured probes were used to obtain depth and completeness of responses. The interview guide was adapted from Guerra et al. [18], and it prompted study participants to reflect on the medical care they had provided to patients in the three months preceding the study interview. Twenty-one questions with and without structured probes were used. We aimed to identify whether providers discuss CCTs as a form of treatment with patients, barriers that the provider identified when discussing CCTs, what kind of patients providers discuss CCTs with, what kind of patients providers do not discuss CCTs with, what aspects within the healthcare system facilitate CCT discussion, perceptions of positive and negative aspects of CCTs, and what strategies they utilize to discuss CCTs. Questions were reviewed by expert advisors within the Implicit Bias Working Group, a group of experts including patients, clinicians, and researchers from diverse backgrounds guiding the ASCO-ACCC Initiative to Increase Racial and Ethnic Diversity in Clinical Trials [17].

To validate the barriers and facilitators elicited during interviews, we used CSR to elicit specific barriers and facilitators of trial discussion during patient–physician encounters. In CSR, a provider uses their own documentation of patient encounters to stimulate the recall of the encounter, and a researcher probes the reasoning behind their medical decision-making [19]. Three to six CSRs are sufficient to provide a reliable and valid assessment of physician performance [20,21]. We discussed 5 encounters with each provider. Providers were instructed to review the chart of the patient most recently seen and continue in reverse chronological, sequential order to maximize recall and prevent cherry picking encounters where trials were recommended. If the provider had no recall of the encounter, that chart was excluded. The investigators and interviewers did not have access to patient health information (PHI) or identifiers. Encounters of patients aged 18 years or older, with a diagnosis of cancer, seen in the previous 1–3 weeks were included. Without disclosing PHI, we requested a 1- to 2-line summary of each encounter, which included the patient’s age, race and ethnicity, reason for visit, and comorbidities. We then asked if a trial a recommendation was made and why or why not.

Interviews were transcribed verbatim and imported into NVivo 12.0 (QSR International). They were read and coded independently by two investigators (CEG and BSC) and then coded jointly using consensus conferences to resolve coding disagreements. Interviews were analyzed using grounded theory techniques [22].

Barriers and facilitators cited were coded and organized around themes and subthemes. We captured the number of participants and instances when a theme was endorsed during the interviews and separately by the CSR.

## 3. Results

### 3.1. Participant Characteristics

We conducted in-depth interviews and chart stimulated recall with 10 practicing oncologists and research coordinators. Five of the 10 participants were female and five were male. Three participants were Asian, three were Black/African American and four were white. Nine of the participants were physicians and one participant was a Clinical Research Coordinator. Six of the participants practiced in academic centers and four in hospitals or health systems. Half of the participants were in the midwest and three were in the south, one in the northeast and one in the western part of the U.S. 

The average age for the patients that were discussed during CSRs was 62 years old, with a median age of 60. Of the 50 patients discussed, 14 of them had breast cancer, 7 pancreatic, 6 esophageal, 5 colon, 5 rectal, 3 lung, 2 prostate, 1 anal, 1 liver, 1 cervical, 1 stomach, 1 small bowel, and 1 vulvar cancer. A total of 25 patients were described as white, 12 as Black, 7 as Asian, and 4 as Hispanic. A total of 23 patients were described as having metastatic disease. In Table 1 we summarize the barriers and facilitators elicited by in-depth interviews and CSR. 

### 3.2. Summary of Findings

Trial discussion rates were very low, occurring in 6 of 50 (12%) encounters. Table 1 shows the list of barriers and facilitators cited during in-depth interviews. Parenthetically, we note the number of times that each barrier and facilitator was cited. In the adjacent column we quantify how many times each barrier and facilitator were elicited and validated by CSR. Data is organized utilizing the four level domains: system, trial, patient, provider. 

#### 3.2.1. Barriers

Below we discuss only the barriers and facilitators that were validated by CSR. Notably, at the system level no cited barriers were validated by CSR. We highlight corresponding insightful quotes from study participants.

#### 3.2.2. Trial Level

Eligibility criteria. Patients can be excluded based on comorbidities. This was the most frequently validated barrier by CSR.

“Some of those exclusions for comorbidities and very strict lab test requirements can unintentionally exclude more patients from minority and underserved populations. That’s not their intent, but that’s their effect”.

Disease factors. No trial available for a specific malignancy.

“I see a lot of rare forms of cancer, and when people ask about clinical trials my answer will be, ‘We don’t have clinical trials and, honestly, no one does because there’s so few cases of this that no one will ever be able to formulate a clinical trial’”.

#### 3.2.3. Provider Level

Assumptions about patients. Relying on data and assumptions when calculating patients’ risks and benefits for trial participation leads providers to not offer trials or mention them briefly.

“The people who maybe I won’t bring it up as frequently with are the ones who initially express, hey, I live too far and I’m not interested if they express initially ‘it’s too much for me to come out’. But if there’s still a trial that comes up I will kind of mention it in passing. They usually have made up their mind”.

One provider described their transparency when weighing the risks and benefits, and then seeing if patients object to their reasoning.

“There are very few people who I wouldn’t approach. There are patients who I’ve mentioned to, ‘There’s a clinical trial that you would be eligible for, but I don’t think it’s a good idea for you right now for these reasons’. I do it that way partly so that if I’m giving more weight to the reasons why I don’t think this is a good idea for them [to see if they] correct me or challenge me on that”.

Inadequate knowledge. Not knowing what CCTs are available on- or off-site and lack of knowledge of new CCTs.

“There’s these other components of the trial that maybe I didn’t go into as much detail with her because it was a brand-new trial to me”.

CCTs are seen as a last treatment option. Providers subsequently do not discuss them unless patients have failed to improve with the standard of care.

“There are perceptions that certainly patients have, but some physicians as well, that trials are the thing you do when there’s nothing else left to do. That doesn’t help”.

#### 3.2.4. Patient Level

Disease factors. CCTs are often not discussed when a patient needs urgent or emergent treatment; the conversation shifts to discussing the most immediate treatment.

“If they need to start treatment immediately and they don’t have time to really consider clinical trials”.

Patient knowledge of CCTs. Patient’s educational background, literacy limitations and lack of knowledge and understanding of CCTs.

Negative attitudes about CCTs. Some underrepresented minority patients are perceived as having more mistrust, skepticism, and negative attitudes about CCTs. The legacy of Tuskegee leads patients to “swiftly” decline CCTs.

“Things like Tuskegee and other incidents in history… the Native American population is another one that hasn’t been offered clinical trials enough, but also there’s a skepticism of when it is offered. I would say that there is even when it’s offered, maybe a swift decline to proceed with discussion, or even after learning, they decline to move forward with it based on ethnicity and background”.

“I can put up a wall due to some of my minority patients in terms of the notions that they don’t want to be experimented on. That’s how sometimes they feel. I’ve had a couple of patients that tell me that, and therefore this wall is up, and I can’t get through it regardless. And there is no further discussion. It’s a big wall. Some of it is due to historical experiences with trials, how they affect minorities in the country”.

Psychosocial factors. When the patient and family are emotionally overwhelmed with a diagnosis of cancer, they do not have the frame of mind to consider additional time and effort required by CCTs.

Culture. A patient’s culture and their perception of medicine can affect the discussion. In some cultures, family may not want the patient to be “experimented on”.

“Especially if the patient is elderly, and their adult children who assume the role of the primary caregiver and primary responsibility for the family might shield their parent. When clinical trials are broached, it’s almost taken as an offense, like ‘you’re going to experiment on my loved one,’ and so the conversation can’t go forward”.

### 3.3. Facilitators

#### 3.3.1. System Level

Larger physical space. A larger space with more rooms to have patient discussions in and to accommodate remaining clinic patients.

#### 3.3.2. Trial Level

CCT availability. Having more available CCTs in each site and for each disease type.

#### 3.3.3. Provider Level

Systematic approach to screening. Systematically screening, reviewing eligibility, and speaking about CCTs to all patients.

“We screen every patient that comes to our center. We have three clinics, radiation oncology and two hematology-oncology. Each of the nurses here is in charge of one of the clinics. We screen all those patients for all our clinical trials. All the patients that come, whether they’re new or they’re follow-up, we continue to screen. If we find a patient that might qualify, we approach the physician, and then we ask the patient”.

Comparison of standard of care vs. investigational therapies.

“I always do it in the context of ‘this is standard of care and then this is investigational’. I present both options. I like to draw it out for them. I compare and contrast what is considered standard of care and what is the clinical trial option for them.

Awareness of CCTs. Being aware of what CCT options are and knowledge of CCTs that a patient may qualify for at other sites.

#### 3.3.4. Patient Level

Disease factors. Patients needing a new treatment either due to disease progression or having stage IV disease.

“At follow up visits if we’re dealing with a metastatic patient, if they have progressive disease I usually will say, ‘we have this trial or, unfortunately there are no trials right now’”.

Referral for CCT or second opinion. Patient is referred to that site for a specific CCT or a second opinion.

Younger age. Younger patients seek CCTs more than older patients.

Motivation and engagement. Patient asking about CCTs.

“If they’re already coming in either knowing about clinical trials or asking to do it, then you’re like, “Okay, great”. It’s less explaining and trying to convince them that it is a potentially better option. It always helps if patients are asking about clinical trials or already interested in clinical trials”.

Earlier introduction to CCTs. Earlier introduction by a provider such as the patient’s surgeon, or by an advocacy organization.

“The biggest issue I find in those populations are, one, mistrust and, two, a lack of understanding of what a clinical trial means. We try to introduce clinical trials earlier in their care. Before they see me, we’ve tried to have the surgeons introduce that. Because people tend to have a bond with their surgeon. If the surgeon is saying, ‘there are things called clinical trials’ or ‘there’s a trial that you should look out for’, that’s one of the things that we tried to do earlier on”.

Positive attitudes: altruism and trust. Specifically, when patients viewed CT participation as beneficial for themselves and for others.

“There were times early in my career where I gave clinical trials a brief mention to someone who had very low education level thinking this is going to go nowhere and they had great questions, were fully engaged, wanted to participate, and saw this as ‘hopefully this will help me, but if not, it will help someone else’. They articulated that altruistic goal of this is a way that I can make something good come from something bad, and that’s powerful. I’ve tried to learn from those experiences and not let that stop me from offering trials”.

## 4. Discussion

Oncologist recommendation is the strongest factor associated with the enrollment of patients in CCTs. If presented with an option to enroll in a CCT, over 50% of patients will enroll with no statistically significant difference in enrollment by race or ethnicity [7]. Yet, little is known about the oncologist’s actual barriers to and facilitators of discussing CCTs. We identified and validated oncologists’ perceived barriers and facilitators for the recommendation of CCTs at the system, trial, provider, and patient levels through a triangulation of methods. By utilizing CSR, we can provide a reliable measure of physician decision making and performance and find the actual barriers and facilitators that participants had regarding the discussion of CCTs with their patients.

No system barriers were validated by CSR. Trial barriers to discussion were the trials’ eligibility criteria and lack of trials for rare diseases. Provider barriers were assumptions (e.g., a trial is too burdensome for patients or patient is not interested in participating), inadequate knowledge about trials, and the idea that trials should be offered as a last resort. Perceived patient barriers were disease factors (e.g., requiring urgent therapy), lack of knowledge, negative attitudes, and psychosocial factors.

A system facilitator was a larger physical space, with dedicated spaces for providers to discuss trials. A trial facilitator was having a greater availability of CCTs. Provider facilitators were having a systematic approach for offering trials, having a system to present the standard of care and trial options in a structured way, and an awareness of trial availability. Patient facilitators were patients with stage IV or progressive disease, those seeking a trial, the absence of comorbidities, young age, the prior knowledge of CCTs, altruism and trust, and motivation and engagement to seek CCTs.

The interview data provided ways to reduce barriers, often through reported facilitators. The lack of CCT availability can potentially be mitigated by being a part of a cooperative group of CCTs [23]. For several decades now, cooperative groups have provided a platform to conduct CCTs in multiple institutions [24]. The Coalition of Cancer Cooperative Groups has created interventions with the goal of facilitating CCT participation nationwide; such tools include the TrialCheck database. The TrialCheck database showcased information regarding available CCTs throughout the country and was integrated into the electronic medical record (EMR) [23]. Though its effectiveness was not determined, participation in cooperative groups could grant providers access to such tools through which the availability and knowledge of new trials can become more prevalent in their clinical practice. Not only can they identify new trials for their patients at the national level, but also they would be able to utilize tools integrated within their respective institution’s EMR that can streamline the process of discussing CCT more frequently by improving accessibility [23].

Similarly, another innovative “Blue Button” clinical trial matching software tool was created by the American Cancer Society that can be integrated into the EMR to help increase access to regional CCTs [25]. TrialTALK aims to address provider barriers to discussing CCTs by providing a framework for cancer treatment discussions [26]. It utilizes a diagram containing the diagnosis, prognostic implications, and available treatments, estimates the efficacy of each approach, and anticipates the impact on quality of life. Though its effectiveness has not been determined, this tool may be helpful in systematically offering CCTs to all patients, and its impact should be studied to standardize CCT discussions by all oncology providers.

Though our study did not focus on the impact of race on provider discussions of CCTs with their patients, implicit bias can also lead providers to perceive minority patients as less ideal study candidates [27]. These findings illustrate that oncology providers need to implement a standardized tool and approach to CCT discussions to mitigate the role of implicit bias in CCT discussions.

Our study shows that providers could overcome assumptions by utilizing a systematic approach to the discussion of CCTs and by establishing a practice policy to prescreen all patients for CCTs. Regardless of their demographics and disease characteristics, patients should be offered a CCT when available, with all treatment options presented side-by-side. Lastly, if assumptions are made, providers should be transparent and check those assumptions with patients to respect their autonomy.

Patient barriers such as their perceived lack of knowledge of CCTs can be mitigated by an earlier introduction to CCTs by team members. Primary care physicians, surgeons, nurses, navigators, peer clinical trial ambassadors, patient advocacy organizations, and the provision of resources including CCT educational videos, websites, and brochures can help address this barrier. However, studies have showcased an interface between medical research literacy (MRL) and clinical trial participation. Participants who were described as having higher MRL were found to be less likely to participate in a clinical trial [28]. Though the exact reason remains unknown, the authors allude to the warranted exploration of the patients’ trust in the medical community. This study illustrates that MRL is not an isolated construct. In addition to medical distrust, our study validated perceived patients’ negative attitudes and psychosocial factors as barriers to discussions of CCTs. These barriers can pose a bigger challenge to understand; therefore, additional analysis is warranted to design interventions to address their role in discussing CCT participation.

The strengths of this study are the triangulation of data, the participants represented a wide range of geographic distribution, and the 50 patients discussed during CSR were diagnosed represented demographic and disease diversity. One limitation of this study is it’s small sample size. A larger study is needed to generalize our findings as well as uncover unidentified barriers and facilitators of CCT discussions. The roles of race and ethnicity and education level were not investigated in this study, but through the analysis of our interviews, education is likely an important factor when conducting CCT discussions and should be formally investigated in a future study. Our study population focused on oncology providers; therefore, it is imperative that future studies focus on collecting and analyzing the perspectives of patients with cancer, to establish what barriers and facilitators lead to their discussion of CCTs with oncology providers. Since we did not have provider rates for CCT discussions with their patients, and our study was not designed tor to have a comparison group. Lastly, since this is a qualitative study, we cannot make conclusions regarding our results’ statistical significance. Nevertheless, to our knowledge, this is the only study that has utilized CSR and used a triangulation of methods to validate stated barriers and facilitators from oncology providers, thereby uncovering the actual barriers and facilitators to CCT discussion. Lastly, we provide strategies which can be formally studied to determine their impact on CCT discussions.

## 5. Conclusions

We identified oncologists’ actual barriers and facilitators for the discussion of CCTs at the system, trial, provider, and patient levels. Many of the identified barriers can be addressed by cited facilitators. Oncology providers should discuss CCTs systematically with all their patients, and care teams should aim to introduce the concept of CCTs earlier. The access and integration of CCT matching systems are a promising strategy to overcome one the main barriers, the availability of local clinical trials, and if effective, should be integrated into clinical practice and become widely available. Future studies should focus on exploring the patient’s actual barriers and facilitators to the discussion of CCT to gain critical insight into the complex variables that affect their conversations and prevent them from receiving the best cancer treatments.

## Figures and Tables

**Table 1:** Barriers and facilitators to cancer clinical trial (CCT) discussion cited during the in-depth interviews, and number of times barrier or facilitator was elicited by chart-stimulated recall.

	Barrier to Discussion of CCT Cited during In-Depth Interview (Number of Participants Who Reported Barrier)	Number of Times Barrier Was Cited during Chart Stimulated Recall	Facilitator to Discussion of CCTs Cited during Interview (Number of Participants Who Reported Facilitator)	Number of Times Facilitator Was Cited during Chart-Stimulated Recall
**System**	Distance to CCT site (2)		Robust CCT workforce: conducting prescreening, consents (9)	
	Time (9)		System that keeps track of patients who providers have discussed CCTs with	
	Lack of a sufficient research workforce		Program integrated into the EMR that identifies clinical trials, reminds providers to discuss CCT and/or access to research team members who can discuss trial.EMR as a tool for matching and communicating (5)	
	Financial toxicity		Being a part of a cooperative group CCT (2)	
	Insurance: out of network restrictions (3)		Larger physical space	1
	Limited visitation policy during the COVID-19 pandemic, less family members helping process CCT discussion		Interpreters, translators (3)	
	Too many CCT options		Chart/checklist with available CCTs	
	Transportation to CCT site		Database that facilitates prescreening	
			Multidisciplinary clinics	
			CCT educational resources: literature, website (3)	
			Principal investigator sending introduction of trials/reminders	
**Trial**	Disease factors: no CCT available for specific malignancy	2	Greater CCT availability (5)	2
	Requirement of additional tissue biopsy		Additional care providers: e.g., another nurse, another clinical trial coordinator involved in patient care	
	CCTs that require frequent visits (2)		CCTs often offer opportunities for more imaging studies	
	Language: lack of availability of consent forms in different languages (3)		Having consent forms in different languages	
	The toxicity of CCT treatments		CCTs provide more treatment options	
	Explaining genomic profiling or biomarker concept to patient		Phase 2 CCTs: (excitement of receiving a new treatment, every patient will receive the treatment)	
	Eligibility criteria (4)	7		
	CCT that has been open for years			
	Treatment drugs that are too new			
	Specific design features: RCT/the concept of randomization, placebo-controlled CCTs, CCTs that are not treatment-based (non-therapeutic clinical trials), correlative studies (4)			
**Provider**	Assumptions (4)	2	Systematic approach to screening (6)	8
	Inadequate knowledge (2)	3	Awareness of CCTs	1
	Forgetfulness: to prepare charts, to do patient prescreening		Comparison of standard of care vs. investigational therapies (2)	1
	The responsibility of discussing CCTs falls solely on the provider		Accountability: leadership or board of directors maintaining providers accountable	
	CCTs are seen as a last treatment option	3	Provider is a researcher	
	Fear of disappointing patients if they are unable participate in CCT		Research staff to remind provider about CCT	
	CCT participation is more difficult than giving standard of care			
**Patient**	Disease factors: urgent or emergent need for treatment	3	Newly diagnosed patient (2)	
	Negative attitudes about CCTs (10)		Disease factors: patient needs a new treatment option (6)	3
	Patient’s comorbidities		Patient is interested in CCTs (4)	
	Language: limited English proficiency (3)		Younger age (2)	1
	Psychosocial	1	Earlier introduction to CCTs (2)	1
	Patient’s poor performance status	1	CCT or second opinion referral (3)	3
	Living far away from CCT site (2)		Good support system (2)	
	Social determinants of health: insurance, finance, transportation (2)		Patient has a good performance status	1
	Culture (2)	1	Lack of comorbidities	2
	CCT participation would stop alternative therapies		Patient adherence	
	Fear of CCT associated side effects		Patient positive attitudes: altruism and trust (4)	1
	Knowledge of CCTs(4)	2	Involvement with advocacy organizations	
	Low patient interest	1	Motivation and engagement (5)	2

## Data Availability

Data supporting the reported results can be shared upon request, as they are not stored in a public access domain.

## Appendix A

### Appendix A.1. In Depth-Interview Questions

Please reflect on the medical care you have provided to your patients in the last 3 months when framing your responses to the questions I am about to ask.

1. Do you discuss clinical trials as a form of treatment with patients?
2. What are the barriers to discussing clinical trials with patients? Any others?

Probe to fully explain answer:

2a. Are there specific types of patients for whom you do not discuss clinical trials? Anything else?
3. What are some factors that facilitate a discussion about clinical trials with patients? Any others? Probe to fully explain answer.
3a. And then at what point in the patient’s care would you normally discuss clinical trials
4. Are there any specific types of patients for whom you do discuss clinical trials? Anything else?
5. What are the things in the health care delivery system in which you practice that help you to discuss clinical trials? Any others?
6. Can you name some positive or negative characteristics or aspects of clinical trials or research that influence whether you do or do not incorporate the topic during your discussion?
6a. Clinical trial/research positive characteristics
6b. Clinical trial/research negative characteristics
7. What are strategies that could help you increase your own rates of discussing clinical trials with patients?

### Appendix A.2. Chart-Stimulated Recall Protocol

1. Inclusion/Exclusion Criteria

For each chart selected, be certain it meets the inclusion and exclusion criteria:

- A patient who was seen in the previous 1–3 weeks
- Patient age >18 or parent/guardian present if ≤18

2. Rating recall of each chart

For each chart selected, rate your recall of the encounter on a scale of 0 to 10 (0 = no recall; 10 = perfect recall). If recall is 0, exclude chart from CSR discussion.

3. Patient summary

If the encounter is recalled, then for each patient, have the physician provide the following information:

- Age
- Race/ethnicity
- Major comorbidities
- Reason for visit

If acute visit: When was the last annual check-up?

- Presence or absence of a discussion about clinical trials at index visit

If yes:

- What was discussed?
- What factors facilitated your discussion of clinical trials?
- How, if at all, did the patient’s race or ethnicity affect your decision to discuss clinical trials?
- Did the patient pursue clinical trial enrollment?

If yes: What trial? When?

If no:

- What factors inhibited you from discussing clinical trials?
- Was there a discussion of clinical trials at any previous visit?

If yes:

- When was that visit? What was the type of visit?
- Did the patient pursue clinical trial enrollment?

If yes: What trial? When?

If no:

- How, if at all, did the patient’s race or ethnicity affect your decision not to discuss clinical trials at this visit and at any previous visit?

Repeat CSR for a total of approximately 5 charts.
